# Refractive Dilemma

**Published:** 2012-01

**Authors:** Alireza Baradaran-Rafii, Mohammad-Reza Jafarinasab, Farid Karimian, Hossein Mohammad-Rabei

## CASE PRESENTATION

**Figure 1.**
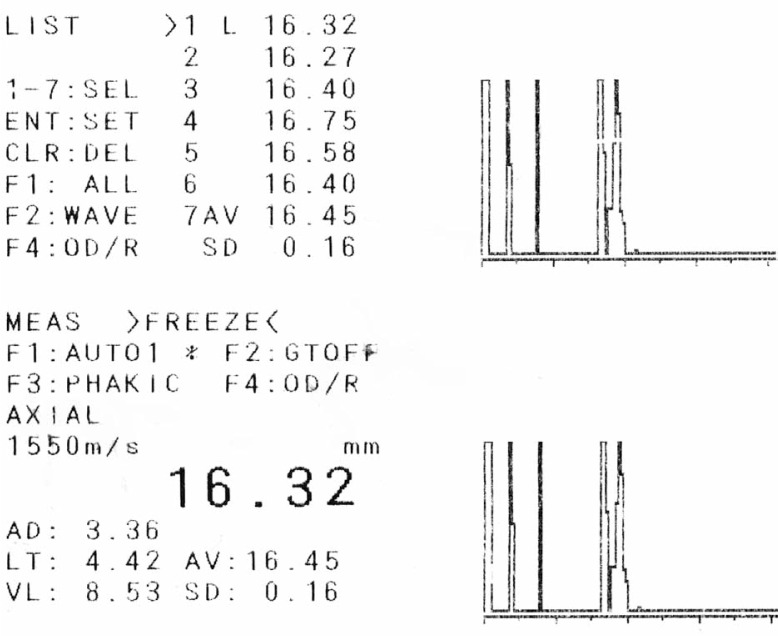
A scan biometry of the right eye

**Figure 2.**
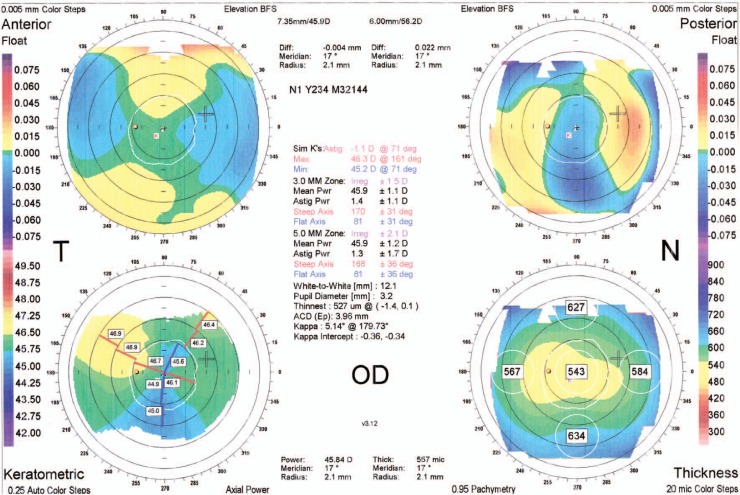
Orbscan of the right eye

**Figure 3.**
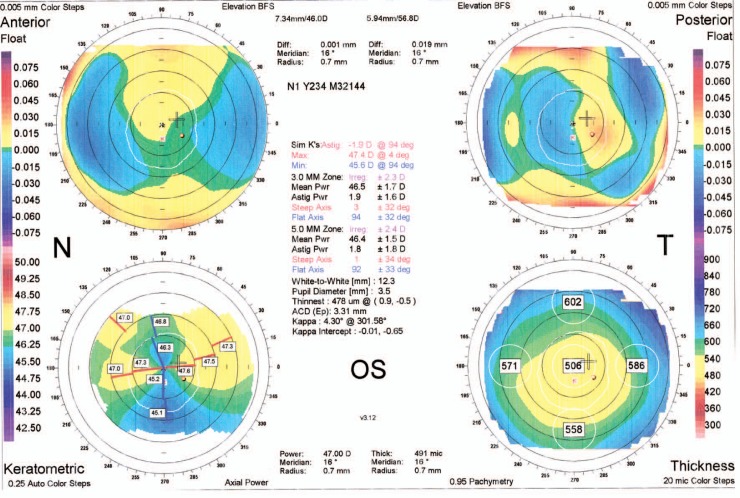
Orbscan of the left eye

A 21-year–old man was referred to an ophthalmology clinic insisting on getting rid of his glasses which he had been using for 11 years. Gross ocular examination was normal except for small eyes. Best corrected visual acuity (BCVA) was 20/40 and 20/50 with +16.00 and +18.00 diopter (D) glasses in his right and left eyes respectively. No ocular deviation was observed and slit lamp and fundus examinations were within normal limits. Figures 1, 2 and 3 demonstrate his A-scan ultrasound biometry and ocular topographic features.

***What is your preferred plan for this patient? Discuss the advantages and disadvantages of the different plans you suggest.***

## Mohammad-Reza Jafarinasab, MD

This patient has high hyperopia associated with amblyopia, slightly more severe in his left eye. Regarding the A-scan in his right eye, short axial length has led to high hyperopia in the context of nanophthalmos. Based on my experience, patients with nanophthalmos can be categorized into anterior and posterior types, both with short axial length. The former group, besides having short vitreous length, have shallow anterior chamber depth (ACD) together with increased lens thickness. In the posterior variant, short vitreous length is the main reason for short axial length, but ACD and lens thickness are normal. Considering the A scan biometry of the right eye, this patient fits into the posterior type category with an ACD of 3.36 mm, lens thickness of 4.42 mm and vitreous length of 8.53 mm which is much less than normal. Nanophthalmos poses higher rates of complications following intraocular surgery as compared to normal eyes; the most dreaded complications such as choroidal effusion and malignant glaucoma are more prevalent in the posterior type while other complications including corneal edema and pupillary block may be more common with the anterior variant. Since this patient is dissatisfied with his glasses, contact lenses are recommended. In the next step, if the patient is intolerant to contact lenses, specular microscopy should be performed. If endothelial cell count is normal, the next step could be an implantable collamer lens (ICL), considering the normal ACD (3.36 mm). Although available ICL powers are less than his level of refractive error; due to young age (21 years) and good accommodative reserve, ICL can dramatically improve his quality of life.

## Farid Karimian, MD

This young man is a case of nanophthalmos with severe hyperopia and moderate amblyopia. Despite good corneal thickness and normal Orbscan findings, excimer laser hyperopic correction is not suitable for hyperopia exceeding +4.00 D. In addition, conductive keratoplasty, which is another option for low hyperopia, is also not applicable to this patient. Although, anterior chamber depth (3.00 mm) is adequate and white-to-white corneal diameter (12.0 mm) is excellent, provided an endothelial cell count of more than 2500 cells/mm^2^, there would be limitations in intraocular lens (IOL) power availability. Phakic intraocular lenses (iris fixated or posterior chamber lenses) cannot correct this high magnitude of hyperopia. The highest refractive error which can be corrected by Artisan-Verisyse phakic IOLs is +7.00 to +8.00 D of hyperopia and hyperopic Artiflex-Veriflex is not available in the market.

The only available option for correction of the extreme level of hyperopia in this patient is refractive lens exchange. The patient should be clearly informed about the advantages and disadvantages of this elective intraocular surgery. Although the risk of retinal detachment is lower than myopic eyes, risks of postoperative endophthalmitis, cystoid macular edema, and uveitis must be considered and explained to the patient prior to surgery.

## Hossein Mohammad-Rabei, MD

The presented findings demonstrate a patient with high hyperopia, normal anterior segment measures and very short vitreous length compatible with nanophthalmos.

Nanophthalmos is a condition in which the eyes are abnormally small without any other ocular defects; nanophthalmic eyes are otherwise normal. On the other hand, in microphthalmia which is rare, besides small eyes, different ocular anomalies such as iris or retinal colobomas are present. The anterior segment is normal in 80% of hyperopic eyes, while it is small in 20%. Characteristics of nanophthalmos are axial length of 14–16 mm and hyperopia of 13–18 D. In patients with small anterior segments, the prevalence of glaucoma is higher and ocular surgery is difficult. Moreover, complications following any type of intraocular surgery are common in nanophthalmic eyes and may result in severe loss of vision.

In these patients, spectacles are among the best choices; they provide relatively good visual acuity at a low cost. In the presented case however, due to high hyperopia and thick glasses, there is 20–30% image magnification, chromatic aberration, image distortion, ring scotoma, peripheral distortion and decreased peripheral field. Since this patient does not wish to continue glasses, contact lenses would be another possibility; regarding the keratometry results (45.00–46.00 D), rigid gas-permeable lenses can be fitted. Contact lenses can decrease image magnification to 7%. They can also decrease other adverse effects of glasses including distortion, chromatic aberration and decreased visual field.

Surgical procedures for correction of hyperopia include external and internal methods. Laser thermal keratoplasty (LTK) using holmium YAG laser is one external procedure applicable for hyperopia less than +3.00 D and therefore not suitable for this patient. Photorefractive keratectomy (PRK), another external surgical technique with a good reputation for correcting myopia and astigmatisim, entails complications such as haze formation, decentered ablation, reduced BCVA and regression. This technique is less suitable for high hyperopia and is normally applied for refractive errors less than +2.00 to +3.00 D. In the last external method, laser in situ keratomileusis (LASIK), creating a large flap on a relatively flat cornea in a small eye is very complicated; moreover, most ophthalmologits apply this technique to correct hyperopias lower than +4.00 D. It can be concluded that, neither PRK or LASIK is appropriate for this patient.

Internal methods include IOL implantation in the anterior nor posterior chamber and clear lens extraction (CLE). Placing phakic IOLs in a highly hyperopic eye is a good option, it preserves accomodation with the possibilty of being replaced. Phakic IOLs have different categories including angle supported, iris claw or ICLs. Image magnification can be further decreased to 4% with the implantation of these lenses. Decrease in endothelial cells, cataract, glaucoma, uveitis and rarely endophthalmitis are among their complications.

**Figure 4.**
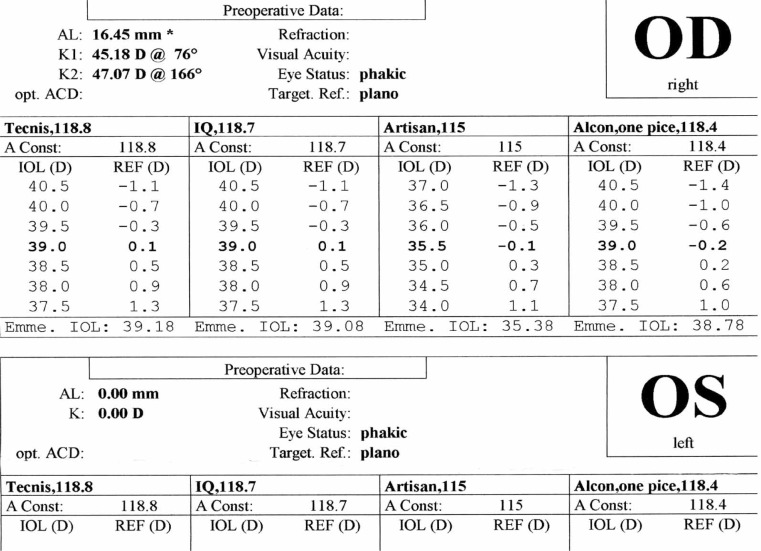
IOL calculation based on SRK II formula

**Figure 5.**
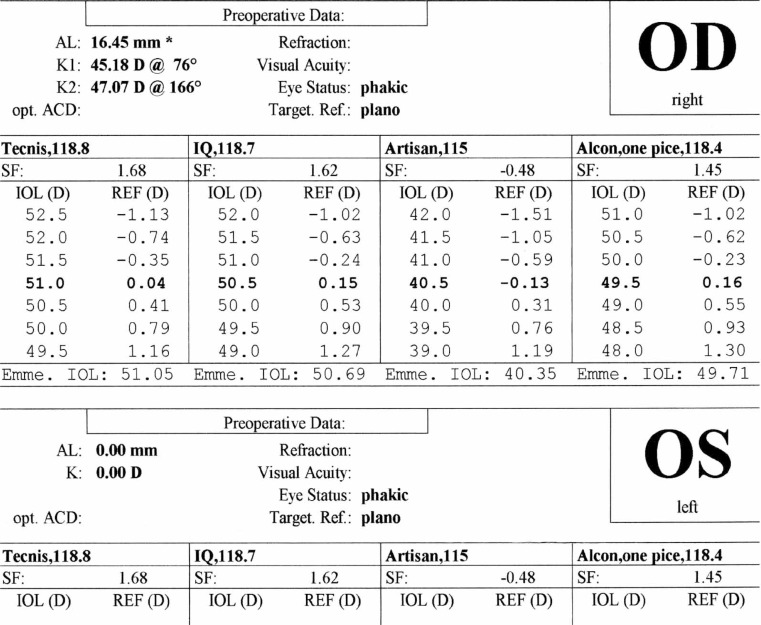
IOL calculation based on Holladay formula

**Figure 6.**
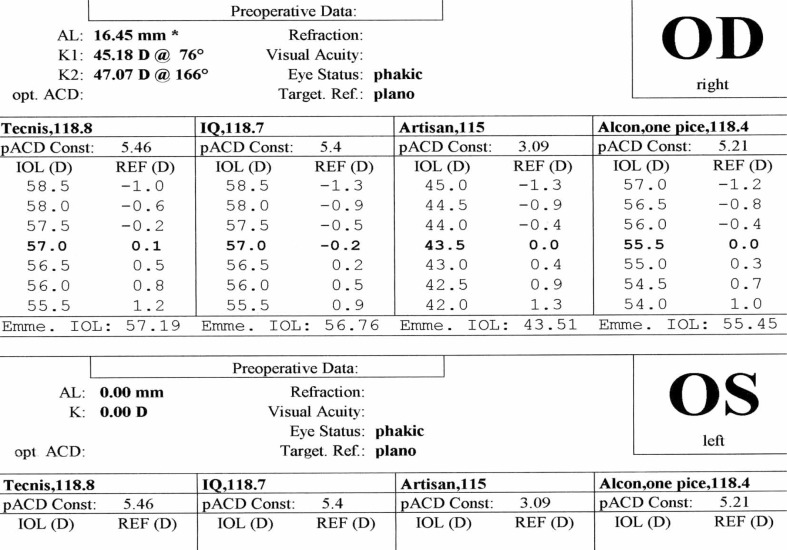
IOL calculation based on Hoffer Q formula

***IOL calculation for the patient’s right eye using different formulas are shown in figures 4, 5 and 6. Given you decide to perform refractive lens exchange for this patient, which method would do you use for IOL power calculation? What power and which formula do you apply? Which type of IOL would you prefer? Which combination of IOL powers would you use? Which power do you implant first and why? What is your preferred site for implantation and why?***

## Mohammad-Reza Jafarinasab, MD

I personally would not recommend refractive lens exchange for this particular patient, however if for any reason the procedure is to be performed, the following points are recommended.

Amblyopia is more severe in the left eye and considering probable intraoperative surgical complications and likely errors in IOL power calculations, surgery would better be performed in the left eye first. This provides an opportunity for better surgical outcomes in the right eye.

For IOL power calculation in this patient, first and second generation formulas such as SRK-I and SRK-II should not be used. Third generation formulas including Hoffer-Q, Holladay-I or Haigis, and preferably fourth generation formulas such as Holladay-II are definitely superior. As observed in the IOL calculation of the right eye, the SRK-II formula may underestimate true IOL power by up to 10.00 D. I personally prefer to apply the Hoffer-Q formula in this patient.

For axial length measurement in nanophthalmic eyes, contact ultrasonic methods should not be used; small underestimations can lead to large errors. Noncontact devices including the IOLMaster, LenStar, immersion ultrasound or preferably mixed methods should be used.

If calculated IOL power does not exceed +40.00 D, a single in-the-bag foldable IOL can be implanted. However in this case with IOL power of +55.00 D, two piggyback IOLs should be used. The first IOL should be placed within the capsular bag and its power should be 30.00–35.00 D. The second IOL can be placed in the bag or in the ciliary sulcus and its power should be 20.00–25.00 D. I, like most surgeons, prefer to implant the second IOL in the sulcus and have reasons justifying this approach. First, the risk of interlenticular opacification with two in-the-bag IOLs is higher as compared to one in-the-bag and one sulcus fixated IOL. Secondly, if any considerable refractive surprise happens, exchange of the second IOL is easier when it is implanted in the sulcus.

A single-piece hydrophobic acrylic foldable IOL would be my first choice for in-the-bag implantation. If available, a model in which 4/5 of the total power is in posterior surface and 1/5 is in the anterior surface (negative shape factor), such as the MA50BM (Alcon, Fort Worth, Texas, USA), would be preferable. The lens for ciliary sulcus implantation should have several features; at least a 6.0 mm optic, total diameter of 13 mm, a rounded anterior optic edge, open loop haptics and 5–10 degrees posterior vaulting. These features provide good centeration and minimal contact with the iris. Usually recommended lenses are the Q-5010V or AQ2010V three-piece silicone IOLs from STAAR surgical. If both IOLs are placed in the bag, due to backward shift on the posterior lens, undercorrection and hyperopia may occur. If the second IOL is implanted in the sulcus, overcorrection and myopia may occur. There is no formula or proposed method to overcome undercorrection. In the case of ciliary sulcus IOL implantation with resultant overcorrection (the preferred method), the following has been proposed. If the power of sulcus IOL is 25.50 to 30.00 D, 1.50 D should be reduced from its power. If the power of sulcus IOL is 15.50 to 25.00 D, 1.00 D should be subtracted, finally if the power of the sulcus IOL is 8.50 to 15.00 D, 0.50 D is reduced. If the IOL power is less than 8.50 D, there is no need to change its power.

## Farid Karimian, MD

Even with the best available IOL power calculation formulas and uncomplicated procedures, the risk of residual refractive errors (overcorrection, undercorrection and surgically induced astigmatism) must be considered. Refractive lens exchange is actually a procedure which may necessitate enhancement by excimer laser, IOL exchange (in the case of postoperative refractive “surprises”) or YAG laser capsulotomy in the future.

Although different combinations of lenses and designs have been introduced for polypseudophakia, my experience with hydrophobic acrylic lenses is excellent. Total calculated power can be divided equally, or 2/3 of the total power can be implanted posteriorly and 1/3 anteriorly. The posterior IOL is implanted into the bag and the anterior one into the sulcus.

The two lenses should not be placed into the bag because there is a risk of interlenticular opacification. Capsulorrhexis must be larger than 6 mm, enabling anterior capsule fusion to the posterior capsule around the optic of the posterior lens, preventing lens epithelial cells from proliferation and migration between the two optics. The posterior IOL should be a single piece design and the anterior one a 3-piece hydrophobic acrylic lens with haptics implanted in the sulcus with 10° angulation. This will press the optics against each other and keep the anterior surface of the sulcus IOL away from the pupil, posterior iris and uveal tissue. A curved optic edge (for example, OptiEdge designed in the Sensar IOL, AMO, USA) on the anterior surface is preferred to reduce rubbing against the posterior iris. Optic diameter for both IOLs must be 6mm and overall diameters should be 13.0 mm helping well centered IOL positioning.

In these cases, especially for “refractive error correction”, IOL power calculation must be very accurate. I usually use three formulas: Holladay-II, Hoffer-Q and SRK-T. None of these formulas is perfect alone. With the aim of optimizing the postoperative refractive outcome as much as possible, the results of these three formulas can be combined. For axial length measurement, immersion biometry and optical coherence techniques such as the IOLMaster or LenStar are preferred.

I usually implant a single hydrophobic acrylic lens first into the bag. At this stage, I completely remove viscoelastic material from the capsular bag and behind the lens and again inject viscoelastics into the sulcus over the in-the-bag optic and into the anterior chamber. I implant the 3-piece sulcus IOL into the sulcus, with haptics perpendicular to the in-the-bag IOL. Viscoelastics must be removed completely and the pupil should be constricted with intracameral acetylcholine. At the end of the surgery, peripheral iridectomy should be performed. This final stage can be performed with a vitrectomy probe.

## Hossein Mohammad-Rabei, MD

One of the most important concerns would be precise measurements especially for angle supported and ICL lenses. For best measurement outcomes, sulcus to sulcus distance should be measured with ultrasound biomicroscopy (UBM) and for their implantation, ACD should be adequate. Specular microscopy should also be requested. Their highest available power is +12.00D which cannot correct the level of hyperopia in this patient; furthermore, this patient is at high risk of glaucoma.

Although clear lens extraction is an easy procedure in a low to moderate hyperope, it is hazardous in patients with high hyperopia or nanophthalmos. Clear lens extraction in patients with high refractive errors provides stable and fast outcomes without regression, however; loss of accommodation may be annoying, especially in young patients.

Intraocular surgery and IOL power calculation in small eyes entails concerns. Even small biometric errors in axial length measurement can lead to high postoperative refractive errors. In addition to IOLMaster which is the best to use, biometry using the immersion ultrasonic technique is also acceptable.

Contact scanning methods are not recommended since probe-cornea contact can create errors in axial length measurement leading to severe postoperative refractive errors. IOLMaster and immersion technique can measure ACD and lens thickness which are important for IOL power calculation. The Holladay-II formula is recommended. Hoffer-Q and Haigis are other acceptable alternatives. Third generation theoretical formulas including SRK-T and Holladay-I may underestimate IOL power leading to residual hyperopia. The reason these formulas underestimate IOL power is that in both of them, effective lens position (ELP) is based on axial length and central keratometric powers. However, in the Holladay-II formula besides central keratometric power and axial lenght, ACD, lens thickness and white to white diameter are considered for ELP. This will reduce errors in this particular patient. In Holladay-I and SRK-T, ELP is closer to the cornea than its actual place leading to residual hyperopia. The Holladay-II formula is not accessible in available A scan machines; the software should be purchased. However, the Hoffer-Q formula is accessible in most machines which is acceptable for IOL power calculation. Hoffer-Q formula should be optimized with the “A” constant of the lens.

In this patient, axial length has been measured with a contact ultrasound machine (probably measurement had been impossible with the IOLMaster). Axial length was at least 16.22 mm and at most 16.76 mm, with an average value of 16.32 mm and acceptable standard deviation of 0.16.

**Table:** 

Formula*	**SRK-II**	**Holladay-I**	**Hoffer-Q**
Power	+39.50	+51.0	+57.00
Predicted refraction	−0.30	−0.24	−0.20

* AC= 118.7

With the first 2 theoretical formulas, measurement error will be high. It is better to use the Hoffer-Q formula which yields IOL power of +57 D. If available, the Holladay-II formula should be considered. Measurement error in Holladay-II and Hoffer-Q are half that of the SRK-II and Holladay-I formulas. IOL power is +57.00 D which is not commercially available. At present, the highest power for hydrophobic acrylic IOLs is +30.00 D and for other lenses is +34.00 D. Theoretically, implantation of an IOL with power of +57.0 D (which is thick and sphere-like) will cause spherical aberrations and distortions, therefore; using 2 thinner lenses (piggyback IOLs) with better optical quality and lower aberration is recommended. This method was first performed by Guyton in 1993.

For piggyback IOL power calculation, it is recommended to add 1.50 D to 2.00 D to the total power because the first lens is pushed backward by the second lens which will create 1.50 D to 2.00 D of hyperopia. This means +57.00(D)+2.00(D)=59(D) in this particular eye. If the Holladay-II formula is applied, there is no need to consider this backward shift since it has already been considered. One +30.00 D or +34.00 D lens is placed in the bag (if available) and the remaining power (+29.00 D or +25.00 D) is implanted in the sulcus after adjusting for sulcus placement as shown in the table below.

**Table:** 

**Sulcus IOL Power**	**Power adjustment**
+25.00 to +30.00	Subtract −1.50 D
+15.50 to +25.00	Subtract −1.00 D
+8.50 to +15.00	Subtract −0.50 D
<+8.00	No change

Therefore, +27.50 D (+29.00−1.50 =+27.50) would be the correct power. Hence, a +30.00 or +34.00 D lens is placed in the bag and a +27.50 or +23.50 D is placed in the sulcus.

In young patients, an acrylic hydrophobic lens with sharp and rectangular edges and preferably negative shape factor is recommended. For sulcus implantation, new generation biconvex silicone IOLs with a rounded edge are recommended. This would reduce the risk of IOL pigment deposition, iris transillumination defects, chronic uveitis and glaucoma. If a silicone lens is not available, a hydrophobic acrylic lens can be used. This will also decrease the probability of interlenticular opacification.

Implantation of 2 IOLs in the bag is not recommended due to the aforementioned complications and backward shift of the first lens and resultant hyperopia. First the nondominant eye should be operated with target refraction of −0.50 to −0.75D. Afterwards, the dominant eye should be operated.

Corneal astigmatism should be considered (1.10 D in the right eye and 1.90 D in the left eye). A 3.2–3.8 mm temporal incision in the right eye will decrease some degree of the against-the-rule astigmatism. In the left eye, a 3.2 mm temporal incision with limbal relaxing incision in the nasal cornea according to Gills nomogram will decrease postoperative refractive errors and result in better uncorrected visual acuity.

In summary, in this young patient with nanophthalmic eyes the best choice is a contact lens and if surgery has to be performed, clear lens extraction along with precautions to decrease complications (glaucoma, choroidal effusion, retinal detachment) and implanting piggyback IOLs can be done. This is appropriate for patients older than 40 years of age and performing it in the presented young patient can be problematic due to loss of accomodation.

